# Establishment and Molecular Phenotyping of Organoids from the Squamocolumnar Junction Region of the Uterine Cervix

**DOI:** 10.3390/cancers12030694

**Published:** 2020-03-15

**Authors:** Yoshiaki Maru, Akira Kawata, Ayumi Taguchi, Yoshiyuki Ishii, Satoshi Baba, Mayuyo Mori, Takeshi Nagamatsu, Katsutoshi Oda, Iwao Kukimoto, Yutaka Osuga, Tomoyuki Fujii, Yoshitaka Hippo

**Affiliations:** 1 Department of Molecular Carcinogenesis, Chiba Cancer Center Research Institute, Chiba 260-8717, Japan; ymaru@chiba-cc.jp; 2 Department of Obstetrics and Gynecology, Graduate School of Medicine, The University of Tokyo, Tokyo 113-8655, Japan; akira2612@gmail.com (A.K.); s.baba.91888@gmail.com (S.B.); mayuyo1976@gmail.com (M.M.); tnag-tky@umin.ac.jp (T.N.); katsutoshi-tky@umin.ac.jp (K.O.); yutakaos-tky@umin.ac.jp (Y.O.); fujiit-tky@umin.org (T.F.); 3 Department of Gynecology, Tokyo Metropolitan Cancer and Infectious Diseases Center, Komagome Hospital, Tokyo 113-8677, Japan; 4 Pathogen Genomics Center, National Institute of Infectious Diseases, Tokyo 208-0001, Japan; yishii@nih.go.jp (Y.I.); ikuki@nih.go.jp (I.K.)

**Keywords:** organoid, uterine cervix, squamocolumnar junction, human papillomavirus, Matrigel

## Abstract

The metaplastic epithelium of the transformation zone (TZ) including the squamocolumnar junction (SCJ) of the uterine cervix is a prime target of human papilloma virus (HPV) infection and subsequent cancer development. Due to the lack of adequate in vitro models for SCJ, however, investigations into its physiological roles and vulnerability to carcinogenesis have been limited. By using Matrigel-based three-dimensional culture techniques, we propagated organoids derived from the normal SCJ region, along with metaplastic squamous cells in the TZ. Consisting predominantly of squamous cells, organoids basically exhibited a dense structure. However, at least in some organoids, a small but discrete population of mucin-producing endocervix cells co-existed adjacent to the squamous cell population, virtually recapitulating the configuration of SCJ in a TZ background. In addition, transcriptome analysis confirmed a higher expression level of many SCJ marker genes in organoids, compared to that in the immortalized cervical cell lines of non-SCJ origin. Thus, the obtained organoids appear to mimic cervical SCJ cells and, in particular, metaplastic squamous cells from the TZ, likely providing a novel platform in which HPV-driven cervical cancer development could be investigated.

## 1. Introduction

The uterine cervix consists of three distinct epithelial types; tall mucin-secreting columnar cells of the endocervix in a single layer, glycogenated stratified squamous cells in the ectocervix, and a transformation zone (TZ) in between, which results from gradual metaplastic replacement of columnar cells by squamous cells during the reproductive age [1]. Reserve cells, putative stem cells in the squamocolumnar junction (SCJ) region, are implicated in this metaplastic process; thereby, their roles have been intensively investigated [2,3]. Whereas the SCJ originally resides at the boundary of the endocervix and ectocervix, the newly formed SCJ is shifted, alongside the extension of the TZ toward the endocervix, to the region connecting the TZ and endocervix. The SCJ and the TZ have been regarded as the most important cytological and colposcopic landmarks in the clinic, based on the fact that the large majority of uterine cervical cancers (UCC) and high-grade squamous intraepithelial lesions (HSIL) arise at this region [4,5]. Whereas human papillomavirus (HPV) is a major cause of neoplastic changes in the cervix for both squamous cell carcinoma (SCC) and adenocarcinoma [6], the incidence of UCC is significantly higher than that of cancers arising from other genital tract tissues [7]. However, the precise mechanisms underlying the predisposition of the cervix toward HPV-driven carcinogenesis have remained elusive.

Recently, a residual embryonic cell population harboring the capacity to differentiate and the vulnerability to undergo neoplastic transformation was documented in both gastro-esophageal [8] and ecto-endocervical junctions [9]. With regard to the uterine cervix, a small discrete population of cuboidal cells in the SCJ region was histologically identified. By micro-dissection and microarray analysis, over 70 genes were identified as upregulated genes by more than two-fold, compared to adjacent squamous or columnar cell populations. In particular, Cytokeratin7 (KRT7), Anterior gradient protein 2 homolog (AGR2), Cluster differentiation 63 (CD63), Matrix metalloproteinase-7 (MMP7) and Guanine deaminase (GDA) were further demonstrated to specifically mark these cuboidal SCJ cells by immunohistochemistry [9]. Intriguingly, all these five markers remained positive in all HPV-related neoplastic tissues and cervix-derived cancer cell lines, but not in the SCC of other tissues in the lower genital tract [9]. Besides, it was demonstrated that SCJ cells give rise to reserve cells [10] and are specific targets of HPV infection in the cervix [11]. These observations point toward the notion that SCJ cells might be highly vulnerable to, and a major cell of origin for, HPV-driven cervical carcinogenesis [12]. As a resource for in vitro studies investigating the relationship between HPV and UCC, several cell lines have been generated. For example, End1/E6E7 and Ect1/E6E7, which are widely used as normal controls for cervical cells originating from columnar cells and squamous cells in the cervix, respectively, were immortalized by the introduction of HPV-derived oncogenes E6 and E7 [13]. Normal immortal human keratinocytes (NIKS) comprise an undifferentiated keratinocyte cell line derived from neonatal foreskin [14] and has been intensively used for the investigation of biological impacts mediated by the introduction of the HPV genome [15]. However, none of these cell lines are, in fact, derived from a discrete population of the SCJ, limiting detailed analysis that focuses on HPV-driven UCC development from SCJ cells.

Organoid culture is an emerging technique that enables the infinite expansion of normal stem cells in culture [16]. It has been applied to various research fields, including infectious diseases [17], developmental biology [18], and tissue regeneration [19]. By taking advantage of propagating normal stem cells in vitro, we have established murine organoid-based ex vivo carcinogenesis models for the intestine [20], lungs [21], hepatobiliary tract [22], and pancreas [23], by in vitro lentiviral gene transduction [24] or chemical treatment [25], followed by inoculation in the dorsal skin of nude mice. More recently, organoid culture techniques have been further applied to patient-derived tumor samples of diverse organs, which revealed that organoids basically retained the histological features and genetic aberrations of the original tumors [26,27,28]. However, there was little progress in their applicability to gynecologic tumors until recently [29], when we established an efficient culture method for ovarian and endometrial tumors [30], by modification of our Matrigel bilayer organoid culture (MBOC) protocol [31], which we previously developed for various murine cells. Moreover, for the first time, we established patient-derived organoids of cervical clear cell carcinoma, a rare type of cervical adenocarcinoma [32], further confirming the validity of the modified culture protocol for gynecologic tumors.

In this study, we aimed to propagate normal cervical cells from the SCJ region by applying our modified MBOC protocol. We successfully expanded HPV-negative SCJ organoids, which were proved to retain many features of the SCJ. These organoids would, therefore, likely contribute to gaining mechanistic insights into how SCJ cells could be deregulated for neoplastic changes.

## 2. Results

### 2.1. Propagation of Patient-Derived Organoids from the Cervical SCJ Region

**Figure 1:**
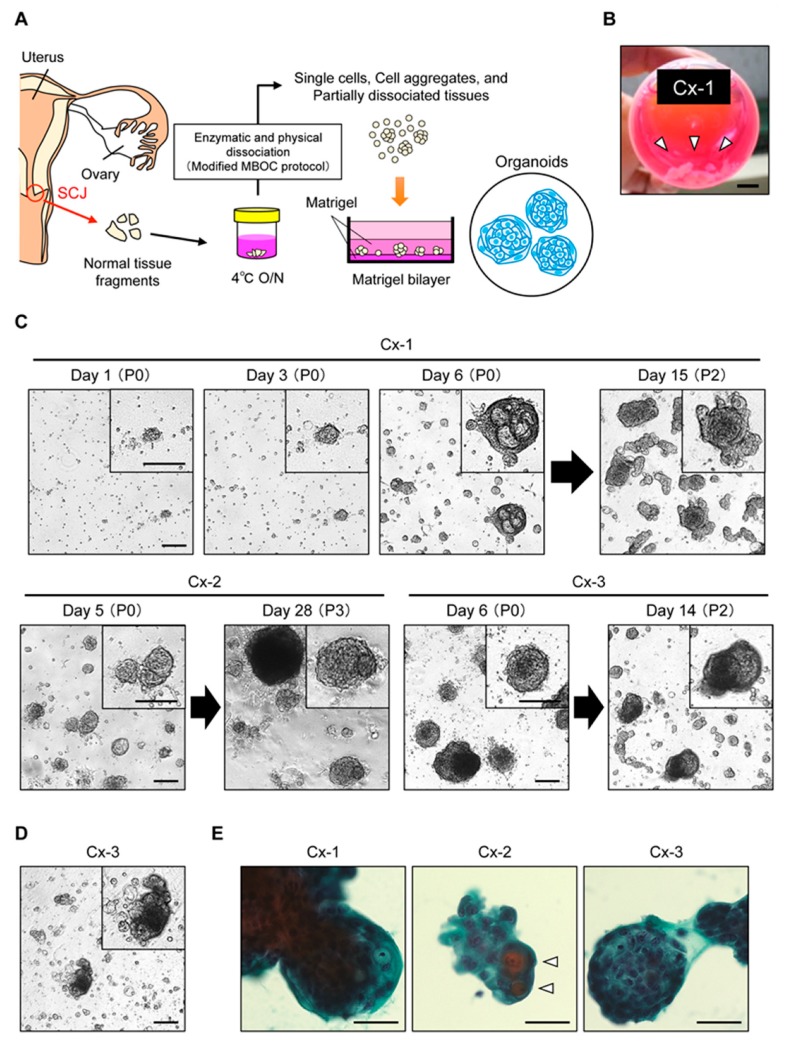
Organoid culture of normal cervical cells derived from the squamocolumnar junction (SCJ) region. (**A**) Schematic presentation of procedures for the establishment of SCJ-derived organoids. A modified Matrigel bilayer organoid culture (MBOC) protocol was used (see Materials and Methods). (**B**) Tissue samples collected from the cervical SCJ. A scale bar indicates 10 mm. White arrowheads depict tissue fragments. (**C**) Phase-contrast images of organoids. Upper panel, representative time-lapse images of SCJ-derived organoids (Cx-1) in the bright field (passage P0 and P2) at 1–6 days and 15 days. Lower panel: representative bright field images of the other SCJ-derived organoids (Cx-2 and Cx-3). Note dense homogeneous cell aggregates in the upper left area of the panel for Cx-2 at day 28. Insets show magnified images of organoids. Scale bars indicate 200 μm. (**D**) Propagation of organoids after cryopreservation. A phase contrast image of Cx-3 organoids at day 14 (passage P1) after thawing is shown. (**E**) Papanicolaou staining of SCJ-derived organoids. Open arrowheads show superficial squamous cells. Scale bars indicate 50 μm.

For future elucidation of the mechanisms underlying UCC development, we set out to conduct an organoid culture of SCJ cells with a modified MBOC protocol [30]. We first tested outpatient biopsy samples targeting the SCJ region (Figure S1A), but we did not achieve robust propagation of organoids. Typical failures included cases where organoids stopped proliferating in early passages or dissociated cells predominantly appeared flat, reminiscent of surface squamous cells (Figure S1B). Based on these observations, we reasoned that collecting tiny amounts of tissue by single biopsy might not be ideal to accurately spot the SCJ and to practically obtain stem or progenitor populations in sufficient amounts. To address this issue, we then selected normal uteri that were surgically co-resected with non-cervical gynecologic tumors. Through careful observation of surface texture, the areas for columnar and squamous epithelium were macroscopically estimated. New SCJ was postulated to be located around their borderline zones. Unlike original SCJ, new SCJ could appear broad in width. Consequently, to ensure collection from the new SCJ region, we comprehensively collected epithelial cells from the areas within a sufficient margin from the estimated SCJ region. Such tissue samples were collected in a hospital immediately after surgery, and processed the next morning following overnight transfer to the lab while maintained in a cold media (Figure 1A). As a culture media, we tested the standard culture media supplemented with EGF, R-spondin-1, Noggin, Jagged-1, and Rho-associated, coiled-coil containing protein kinase (ROCK) inhibitor Y27632, which we have confirmed robustly applicable to organoid culture of murine primary cells from various organs and human gynecological neoplasms [20,22,23,30,32], to facilitate future cross-referencing of organoids from various organs.

We tested SCJ samples from four independent patients (Table 1). Dissociated cells were plated onto solidified Matrigel and subjected to 3D culture. Organoids kept proliferating for at least one month over several passages until we ceased the culture. For example, organoids Cx-1 (Figure 1B) derived from Patient #1, initially exhibited a round morphology with dense and cystic features, but grew in an irregular shape with multiple budding or chain-like structures after several passages (Figure 1C). Similar results were obtained for Cx-2 and Cx-3 from Patients #2 and #3, respectively, while some organoids became resistant to enzymatic and physical dissociation, forming dense homogeneous cell aggregates, as seen in Cx-2 (Figure 1C). Since these observed features are not common to the usual organoids from the intestine [33] but from those of the esophagus [34], we speculated that squamous cell differentiation might be prominent in organoids. With regard to Cx-4 from Patient #4, the majority of the cells appeared to be differentiated squamous surface cells, as frequently observed in biopsy samples, and resulted in a gradual decline of an actively proliferating population, suggesting that the SCJ was not properly collected in this case. A total of 31 HPV genotypes proved negative in the three propagated organoids (Table 1). Besides, organoids were feasible for highly efficient lentiviral gene transduction (Figure S2) and tolerated cryopreservation (Figure 1D). These observations strongly suggested that normal SCJ cells were established for studies to reconstitute UCC development ex vivo. In Papanicoloau staining, a routine procedure for cervical smear samples in the clinic, organoids Cx-1 and Cx-3 appeared to exhibit a solid nature with a layer of cuboidal or thin epithelial cells on the surface, and orange-colored flat cells were observed in Cx-2 organoids, both suggestive of the presence of terminally differentiated squamous cells (Figure 1E).

### 2.2. Mutually Exclusive Localization of Ectocervix-Like Cells and Endocervix-Like Cells within Organoids

**Figure 2:**
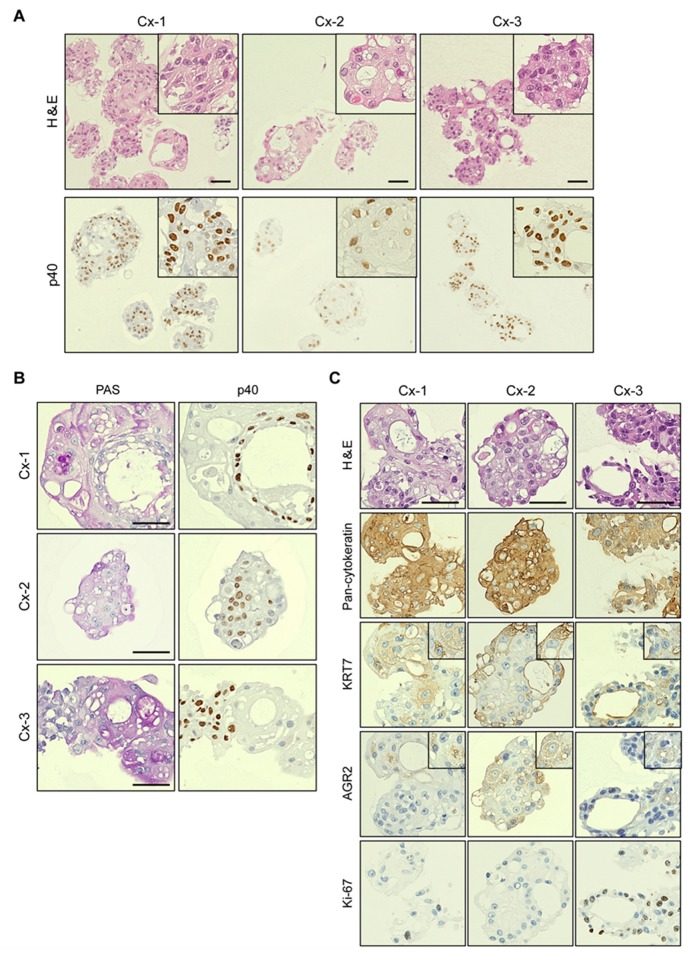
Histological characterization of SCJ-derived organoids. (**A**) Histological examination of thin sections. Organoids Cx-1 to Cx-3 were analyzed. Upper panel, hematoxylin and eosin (H&E) staining. Lower panel, immunostaining for the squamous cell marker p40. Insets show magnified images. Scale bars indicate 50 μm. (**B**) Characterization of cell lineages in SCJ-derived organoids. Serial sections of organoids were immunostained for p40 and by Periodic acid-Schiff (PAS) reaction. PAS reaction visualizes mucins produced by endocervix cells. Note that each staining shows a reciprocal pattern. Scale bars indicate 50 μm. (**C**) Expression of SCJ markers in organoids. Serial sections of organoids were histologically analyzed. H&E staining and immunohistochemical staining are shown. Scale bars indicate 50 μm. Insets show magnified images.

With thin sections of formalin-fixed and paraffin-embedded (FFPE) samples, we found that organoids basically displayed dense structures (Figure 2A). Although cavity-like structures or cysts were occasionally observed within organoids, they tended to lack the regular lining of cells with apico-basal cell polarity, unlike typical cystic structures seen in columnar cell organoids (Figure 2A). Immunostaining for the squamous cell marker p40, the delta N isoform of p63, revealed that most cells, if not all, were positively stained throughout organoids (Figure 2A). This observation is in line with the notion that collected samples would contain TZ/squamous cells in large quantities. On the other hand, given that the SCJ region resides in the interface between TZ/squamous cells and columnar endocervix cells, the propagated organoids are supposed to contain endocervical cells as well. However, cystic structures reminiscent of columnar epithelial cell features were not particularly evident and those few cystic structures were invariably p40-positive in organoids (Figure 2A). To facilitate identification of cells with endocervical differentiation, we conducted Periodic acid-Schiff (PAS) reaction for staining organoids, anticipating that visualized mucins would serve as a surrogate marker of endocervical differentiation. Whereas intense staining was detected only in a subset of organoids (~10%), its distribution invariably showed a reciprocal pattern to that of p40 (Figure 2B). Indeed, p40-positive cells were circumferentially lined along cystic structures as in Cx-1, concentrated in the middle as in Cx-2 or exclusively along one half of an organoid, as in Cx-3, whereas PAS-positive cells were all p40-negative (Figure 2B). After careful histological examination of many organoids, we concluded that the staining patterns of p40 and PAS were, indeed, mutually exclusive, with no intermediate cells, such as both positive or both negative, for p40 and PAS staining.

The co-existence of two distinct cell populations in a back-to-back manner within single organoids greatly resembles the configuration of the actual SCJ, which prompted us to ask whether previously described cuboidal SCJ cells [9] could also be identified in the organoids. As KRT7 and AGR2 have been shown to specifically mark cuboidal SCJ cells by immunohistochemistry [9], we examined expression of these two proteins in organoids. In all three cases, a pan-cytokeratin antibody targeting epithelial cells diffusely stained organoids, while KRT7 was only focally detected (Figure 2C). Similarly, AGR2 was also focally detected, albeit to a lesser extent in terms of the stain-positive area (Figure 2C). With regard to Cx-2 organoids, serial sections were subjected to PAS staining and immunostaining, which clearly indicated that SCJ marker-positive cells appeared to coincide with PAS-positive cells (Figure 2B,C). These observations suggest that SCJ cells might be present in organoids, but only as a fraction of PAS-positive cells. Given the absence of markers highly specific to each population, however, endocervical cells and SCJ cells were indistinguishable. There were only a few cells that were positive for Ki-67, but their localization did not seem to be correlated with that of SCJ marker- or p40-positive cells (Figure 2C).

### 2.3. Many SCJ Markers Exhibited Higher Levels of Expression in Organoids than in Non-SCJ Cervical Cell Lines

**Figure 3:**
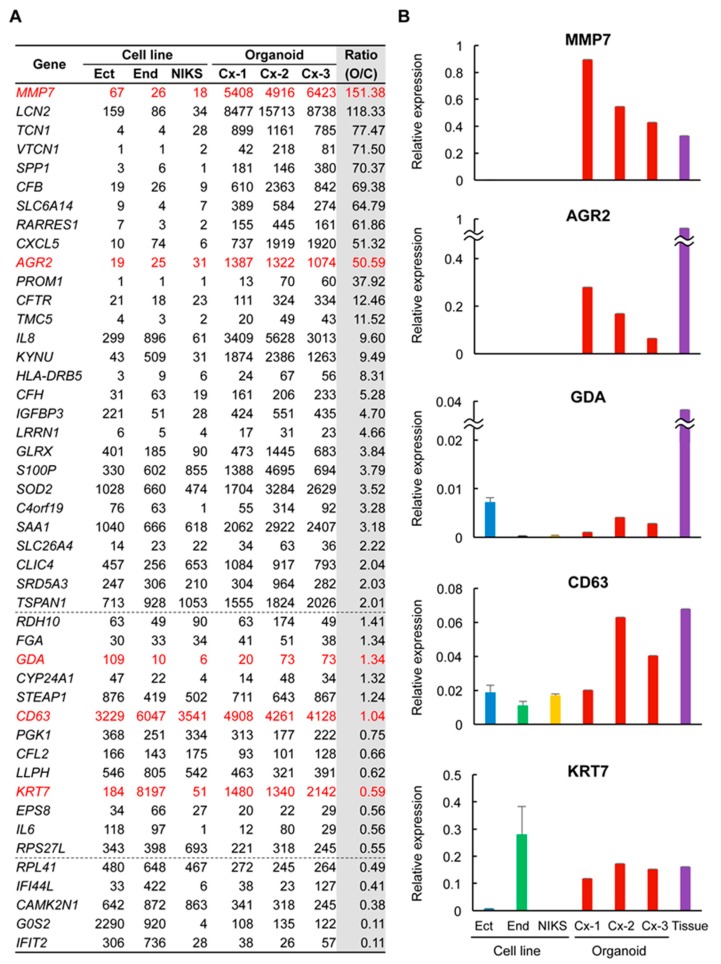
Expression profiles of putative SCJ cell markers in SCJ-derived organoids. (**A**) Expression levels of SCJ marker genes. Microarray analysis of non-SCJ-derived immortalized cell lines: End, Ect, normal immortal human keratinocytes (NIKS), three SCJ-derived organoids Cx-1 to Cx-3, and one SCJ tissue sample. Five commonly used SCJ markers are highlighted in red. Ratio of mean signal intensity for cell line and organoids was calculated. Dashed lines indicate two-fold and 0.5-fold changes. (**B**) Validation of microarray data by RT-qPCR. Expression of the five SCJ markers was examined.

To gain insights into the extent to which the organoids functionally reflect the properties of SCJ cuboidal cells, we next performed microarray analysis of the organoids. Immortalized cervical cell lines Ect1/E6E7 and End1/E6E7 [13] were analyzed as normal references representing ectocervix cells and endocervix cells, respectively. NIKS, undifferentiated keratinocytes [14], were also included as a reference in light of their frequent use in HPV infection experiments. Of 77 genes proposed as SCJ markers [9], 12 genes did not match the corresponding probes in the microarray used in this study (Table S2). Among the remaining 65 genes, 17 genes only had low expression throughout all samples in this study (Table S1). Twenty-eight genes had more than a two-fold expression level in the organoids compared to the three cervical cell lines (Figure 3A). Notably, 13 genes, including MMP7 and AGR2, had more than a 10-fold upregulation in organoids. The expression level was similar and lower in the organoids in 13 and five genes, respectively. GDA, KRT7, and CD63 were not necessarily upregulated compared to non-SCJ cell lines (Figure 3A). These results strongly suggest that the organoids might retain most, if not all, expressions of many SCJ markers, strongly suggesting the superiority of organoids to non-SCJ cervical cell lines as a model of SCJ cells.

For five representative SCJ markers previously well characterized in immunohistochemistry [9], we performed RT-qPCR to validate the microarray data. Whereas we included one primary SCJ tissue sample, all five SCJ markers were expressed in the primary SCJ tissue sample in RT-qPCR analysis, confirming their validity as SCJ marker genes. The expression levels of MMP7 and AGR2 were strikingly higher in all the organoids compared to those in the cell lines (Figure 3B). Although the organoids abundantly expressed KRT7 and GDA, their high-level expression was also observed in End1/E6E7 and Ect1/E6E7, respectively, questioning the specificity of these SCJ markers. Besides, all organoids and cell lines expressed CD63 at similar levels, also negating its specificity for SCJ cells. These results were essentially consistent with the microarray data, suggesting the validity as a transcriptional profile. In addition, these observations also pointed toward the notion that, among 77 genes previously proposed as SCJ markers, a subset of genes, including MMP7 and AGR2, might be SCJ cell markers with the highest specificity and sensitivity that can be expressed, even in an in vitro setting, in a cell-autonomous manner.

Now that we had established that SCJ cells would likely reside in organoids, we asked if reserve cells, putative stem-like cells in the cervix and those implicated in squamous metaplasia [10], could also be detected in organoids. We conducted immunostaining of organoids for reserve cell marker KRT17, to unexpectedly find that it was diffusely expressed in nearly all cells throughout organoids for Cx-1, Cx-2, and Cx-3 (Figure S3A). Moreover, a similar level of KRT17 expression was observed in microarray analysis for all non-SCJ-derived cell lines (Figure S3B). Considering that KRT17 also marks squamous metaplasia and immature types of cells [35], we supposed that the observed high level and ubiquitous expression of KRT17 in vitro might not be informative in spotting reserve cells, but rather might reflect the metaplastic and undifferentiated status of the cell lines and organoids, respectively.

### 2.4. Genes Related to Inflammation and Immune Response Were Highly Expressed in Organoids

**Figure 4:**
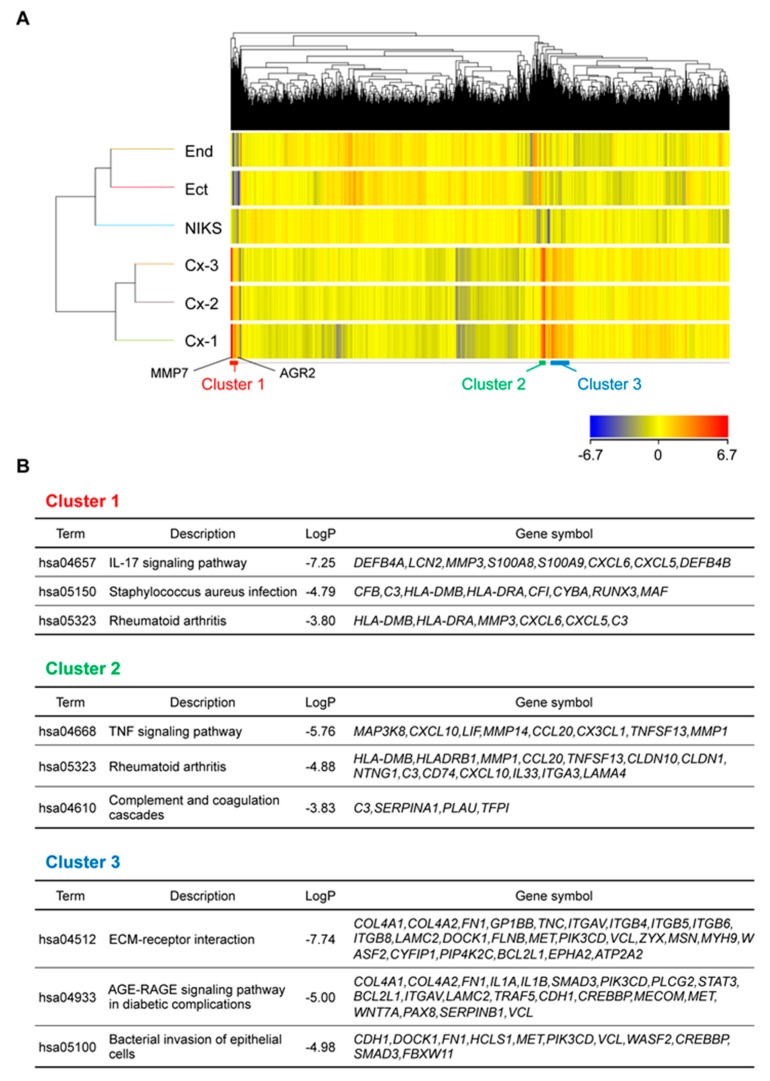
Transcriptome analysis of SCJ-derived organoids. (**A**) Heat map for two-way clustering analysis. SCJ-derived immortalized cell lines: End, Ect, and NIKS, and three SCJ-derived organoids Cx-1 to Cx-3 were analyzed. Note that MMP7 and AGR2 were included in Cluster 1. (**B**) Clusters of specifically upregulated genes in SCJ-derived organoids.

To further explore common features of organoids in an unbiased manner, we conducted a two-way hierarchical cluster analysis on microarray data. Intriguingly, the organoids displayed highly similar gene expression patterns and segregated as a cluster (Figure 4A), although they were derived from different patients. We focused on three gene clusters, Clusters 1–3, in which gene expression levels were significantly higher in the organoids than in the cell lines. KEGG pathway analysis was conducted based on the genes included in each cluster. Genes related to inflammatory reactions, such as the IL-17 signaling pathway, TNF signaling pathway, and rheumatoid arthritis pathway, were significantly enriched in Clusters 1 and 2. Among the SCJ cell markers most upregulated in organoids, MMP7, AGR2, LCN2, CXCL5, and CFB were co-segregated in Cluster 1, raising the possibility that transcription of SCJ markers might be commonly regulated as a result of the activation of specific pathways. On the other hand, genes related to ECM-receptor interaction were significantly enriched in Cluster 3 (Figure 4B), which might reflect the presence of Matrigel in the organoid culture.

## 3. Discussion

No SCJ-derived cells in culture have been documented to date, despite their relevance in HPV infection to the cervix and subsequent carcinogenesis. In this study, we demonstrated that normal SCJ samples from patients could give rise to organoids that are robustly propagated and exhibit many features of cuboidal SCJ cells. Intriguingly, they specifically expressed many SCJ markers and comprised dual cell lineages, with many squamous cells resembling TZ. Moreover, unlike widely used cervical cell lines, long-term culture was feasible in this study, even without immortalization by introduction of HPV-derived E6 and E7 oncogenes. These organoids are therefore likely the first in vitro model for normal SCJ cells under physiological conditions. Whereas 77 genes were originally reported to be specifically upregulated in SCJ cells [9], we confirmed through transcriptome analysis that only a subset of genes in fact showed higher levels of expression in organoids compared to those in non-SCJ cell lines. Notably, of five representative SCJ markers in immunohistochemistry, only two (MMP7 and AGR2) were specifically upregulated in organoids. We, therefore, assume that these 77 genes need to be curated by thorough comparison with genes specifically upregulated in SCJ-derived organoids, as revealed in this study. In microarray and qPCR analyses, KRT7 and GDA were highly expressed in End1 and Ect1, respectively. These observations suggest that KRT7 and GDA might be broadly expressed in cells with endocervical and ectocervical differentiation, respectively, and that these immortalized non-SCJ-derived cell lines might likely retain the features of their original tissues. In this regard, SCJ markers superior to the five molecules currently used might well be identified based on this study.

We found that the obtained organoids were mostly solid and showed a significant bias toward squamous differentiation. Considering that the SCJ region harbors reserve cells that drive squamous metaplasia, we initially assumed that reserve cells would be present in SCJ-derived organoids and yield metaplastic squamous cells, leading to the reconstitution of a TZ equivalent in organoids. Naturally, we aimed to detect reserve cells in organoids by immunostaining for a reserve cell marker KRT17. However, nearly all the cells in organoids were positively stained for KRT17, making it impossible to pinpoint reserve cells. As KRT17 is also highly expressed in squamous metaplasia, these observations might rather reflect the predominant presence of metaplastic cells, mimicking the TZ in organoids. Future development of reserve cell-specific markers will clarify KRT17′s localization in organoids. Another feature of the SCJ region is that it accommodates two distinct cell populations with differentiation into ectocervix/squamous cells and endocervix/columnar cells. We adopted PAS to readily identify mucin-secreting columnar cells in organoids with mostly squamous cells. Whereas PAS staining is generally used for detection of glycogen in the surface and intermediate layer of squamous epithelium, or mucin in the glandular epithelium, PAS-positive cells in this study were confined to a p40-negative non-squamous cell population within organoids. Hence, it is likely that PAS-positive cells in the organoid would represent an endocervix cell-like population. Since PAS-positive cells largely overlapped with a population positively stained for SCJ markers, SCJ cells in organoids might be present as PAS-positive cells, which could, in turn, give rise to reserve-like cells. Given the lack of highly specific markers, it is currently difficult to distinguish between endocervix-like cells and SCJ cells solely by histological approaches.

The co-existence of a dual cell population of distinct lineages in a single organoid strongly suggests that SCJ cells gave rise to both populations. However, there is a possibility that a similar observation can be alternatively achieved. One is that squamous cells and endocervix cells have their own progenitor cells, which can propagate on their own, but which coincidentally aggregate during subculture to form single organoids. Given that PAS-positive cells were detected only in a subset of organoids, the presence of organoids genuinely consisting of squamous cells cannot be completely ruled out. Nonetheless, since PAS was only focally stained in any PAS-positive organoids, we speculate that the seeming lack of PAS-positive cells across organoids in many cases might be simply attributable to examination on cross-sections. Whole organoid-basis analysis, such as flow cytometry, might be able to address this issue, given the future development of highly specific markers that distinguish endocervix cells from SCJ cells. We also assume that the presence of endocervix cell-only organoids would be unlikely, because there were no such organoids in any cross-section. The other possibility is that differentiated squamous cells and endocervix cells derived from the original SCJ cells coincidentally attached to each other to form hybrid organoids as we observed. We presume that this is also unlikely, because we learned that differentiated squamous cells in the clinical specimens were extremely difficult to proliferate in organoid culture conditions. Collectively, it is likely that the new SCJ, along with the TZ, was functionally reconstituted in organoids, although it remains to be confirmed by further investigations, such as single cell transcriptome analysis. It should be also noted that, as a limitation of this study, SCJ-derived organoids were characterized only in terms of expression markers and histological features. Functional evaluation of organoids and optimization of culture conditions are to be pursued, in order to further solidify the authenticity of the organoids. By making these efforts, the mechanisms of how estrogens could induce development of TZ, for example, might be clarified in a future study.

Previous HPV infection studies have mainly utilized NIKS as a host and provided enormous knowledge on HPV-dependent transformation of keratinocytes and, thereby, SCC development. Although the implications from these studies are still valid in terms of the relationship between HPV and keratinocytes, it is unclear whether the results could be extrapolated to SCJ-originating UCC development, as it is highly vulnerable to HPV infection and subsequent carcinogenesis, unlike keratinocytes. Now that we have established SCJ-derived organoids, investigations on cervical carcinogenesis by using more common and susceptible cells of origin would pave the way to gaining more detailed insights into HPV-driven UCC development. It is also tempting to speculate that high expression level of genes related to inflammation immune response might underlie the high affinity of HPV to SCJ cells. Synchronous transcriptional regulation across organoids from different patients suggests that there might be a common upstream regulator for SCJ cells, which could also serve as a specific stem cell marker. By using these organoids, exploration of such factors will be warranted.

## 4. Materials and Methods

### 4.1. Patient Information

Normal cervical tissues were obtained from four patients who underwent hysterectomy in the University of Tokyo Hospital. None of the four patients had any malignant findings on the cervix. They had regular menstrual cycles and no episode of hormonal treatment for at least 6 months before hysterectomy. Menstrual cycle was determined by histological examination. HPV status was determined by genotyping of organoids. The Ethics Committee of The University of Tokyo Hospital (approval number 12017) and Chiba Cancer Center (approval number H30-216) approved all experimental procedures in this study. Written informed consent was obtained from all the patients.

### 4.2. HPV Genotyping

HPV genotyping assays were performed by PCR with PGMY primers followed by reverse line blot hybridization, as previously described [36]. This assay can detect 31 HPV genotypes, including HPV 6, 11, 16, 18, 26, 31, 33, 34, 35, 39, 40, 42, 44, 45, 51, 52, 53, 54, 55, 56, 57, 58, 59, 66, 68, 69, 70, 73, 82, 83 and 84.

### 4.3. Isolation and Organoid Culture of SCJ Cells

A median section in the anterior wall of the resected uterus, from the endocervical canal to the fundus, was obtained immediately after surgical resection. Through careful observation of the surface texture of the uterine mucosa, the areas for columnar and squamous epithelium were roughly estimated. The SCJ region was postulated to be located around their borderline zone. SCJ samples were obtained with scissors, from almost the entire area around the postulated SCJ region, approximately 2 cm width and 2–3 mm depth, except for the median part of the posterior wall, so as not to interfere with pathological diagnosis. Such samples were collected in a hospital immediately after surgery and preserved overnight at 4 °C in Advanced DMEM/F12 (Thermo Fisher Scientific, Waltham, MA, USA). The next morning, SCJ samples were further dissociated into cell aggregates or single cells by enzymatic digestion with 2 μ/mL dispase II, 1 mg/mL collagenase P (Roche Diagnostics K.K., Tokyo, Japan) and Accumax (Innovative Cell Technologies, San Diego, CA, USA). Primary organoid culture was conducted according to the modified MBOC protocol, as previously described [30]. Briefly, resuspended cells were plated on solidified Matrigel (BD Biosciences, Franklin Lakes, NJ, USA). The following morning, viable cells attached onto Matrigel were covered with Matrigel and overlaid with media to start the organoid culture. Organoid culture media was advanced DMEM/F12 (Thermo Fisher Scientific) supplemented with 50 ng/mL human EGF (Peprotech, Rocky Hill, NJ, USA), 250 ng/mL R-spondin1 (R&D, Minneapolis, MN, USA), 100 ng/mL Noggin (Peprotech), 10 μM Y27632 (Wako, Osaka, Japan), 1 μM Jagged-1 (AnaSpec, Fremont, CA, USA), L-glutamine solution (Wako), penicillin/streptomycin (Sigma-Aldrich, St. Louis, MO, USA), and amphotericin B suspension (Wako). pCDH-CMV-MCS-EF1-copGFP (System Biosciences, Mountain View, CA, USA) was introduced into organoids as a green fluorescent protein (GFP)-expressing vector, as previously described [24].

### 4.4. Pathological Analysis

Following de-polymerization of Matrigel with Cell Recovery Solution (BD Biosciences, Franklin Lakes, NJ, USA), organoids were collected at day 14 (P2) for Cx-1, d21 (P2) for Cx-2, and d10 (P1) for Cx-3, followed by resuspension in iPGell (GenoStaff, Tokyo, Japan). The iPGell-embedded organoids were fixed in 10–15% buffered neutral formalin, dehydrated and embedded in paraffin. FFPE samples were sectioned at 3 μm thickness and stained with hematoxylin and eosin (H&E). Periodic acid-Schiff (PAS) reaction was conducted to visualize mucin production. Dako Autostainer Link48 (Agilent, Santa Clara, CA, USA) was used for automatic immunohistochemical (IHC) staining with the following primary antibodies; KRT7 (clone OV-TL 12/30, Thermo Fisher, 1:100), AGR2 (clone D9V2F, Cell Signaling Technology, 1:800), pan-cytokeratin (clone AE1/AE3, Abcam, 1:40), Ki-67 (clone MIB-1, Dako, ready-to-use). The following primary antibodies were used for manual staining: p40 (clone BC28, Abcam, 1:40), Cytokeratin 17 (clone E-4, Santa Cruz, 1:250). The reactions were visualized with the Dako REAL EnVision Detection System (DAKO, Glostrup, Denmark) using diaminobenzidine chromogen as the substrate. For cytology, organoids were immediately fixed in 95% ethyl alcohol and subjected to Papanicolaou staining.

### 4.5. Cell Lines

Ect1/E6E7 (ATCC CRL-2614^TM^, Manassas, VA, USA) and End1/E6E7 (ATCC CRL-2615^TM^) were maintained as previously described [13]. Normal immortal human keratinocytes (NIKS, ATCC CRL-12191^TM^) were cultured in F medium in the presence of mitomycin C-treated NIH3T3 cells, as previously described [14].

### 4.6. Microarray Analysis

Total RNA of cell lines and organoids was extracted using an RNeasy Mini Kit (QIAGEN, Hilden, Germany), at day 14 (P2) for Cx-1, d25 (P3) for Cx-2, and d10 (P1) for Cx-3. These RNA samples were subjected to the TORAY 3D-gene analysis service using the 3D-Gene Human Oligo chip 25K (TORAY, Tokyo, Japan). Total RNA was amplified and labeled with Cy5, then hybridized with a 3D-Gene chip. Signals were detected on a 3D-Gene scanner (TORAY) and normalized according to a global normalization method in which the median value of the detected signal intensities was adjusted to 25. With regard to SCJ markers, genes without corresponding probes and with an average signal intensity lower than 20 are listed in Table S2. Differentially expressed genes between three cell lines and organoids were extracted from the microarray data. Genes with normalized signal intensities less than 20 in more than three samples were excluded from the analysis. GeneSpring GX (Ver.14.9.1, Agilent Technologies, Santa Clara, CA, USA) was used to generate a heat map. Metascape [37] was used to identify enriched KEGG pathways of the focused clusters. A *p*-value < 0.05 was considered statistically significant. Microarray data were deposited to GEO accession: GSE138554.

### 4.7. RT-qPCR

Extracted RNA was reverse transcribed using ReverTra Ace^®^ qPCR RT Master Mix (TOYOBO, Osaka, Japan) according to the manufacturer’s instructions. To assess mRNA expression of GAPDH, KRT7, AGR2, MMP7, CD63 and GDA, qRT–PCR was performed using a Light Cycler 480 (Roche Diagnostics, Mannheim, Germany). Relative expression to GAPDH is shown. PCR reactions were conducted in triplicate and means ± SD are shown. Primer sequences and annealing temperatures are listed in Table S2.

## 5. Conclusions

In conclusion, normal cervical organoids were established, which exhibited features of SCJ cuboidal cells and TZ metaplastic cells. To the best of our knowledge, this is the first demonstration of stably propagating primary SCJ cells. As an in vitro model for cell of origin in UCC development, these organoids would likely become a useful resource relevant for the elucidation of multi-step processes of HPV-dependent cervical carcinogenesis.

## Figures and Tables

**Table 1:** Summary of clinicopathological features of the patients.

Patient	Organoid	Age	Parity	Menstrual Cycle	Disease	HPV
#1	Cx-1	53	0	Proliferative phase	Ovarian cancer	Negative
#2	Cx-2	33	0	Secretory phase	Ovarian borderline tumor	Negative
#3	Cx-3	40	0	Secretory phase	Ovarian cancer	Negative
#4	Cx-4	50	0	Not available	Uterine body tumor	Not tested

Abbreviation: HPV, human papillomavirus

## Supplementary Materials

The following are available online at https://www.mdpi.com/2072-6694/12/3/694/s1, Figure S1: A failed case of organoid culture of biopsy samples, Figure S2: Highly efficient gene transduction of SCJ-derived organoids, Figure S3: KRT17 expression in organoids, Table S1: SCJ marker genes whose expression was not verified in organoids, Table S2: Primers for RT-qPCR.
