# Supplementary material for: Establishment and Molecular Phenotyping of Organoids from the Squamocolumnar Junction Region of the Uterine Cervix
Source: Cancers (Basel). 2020 Mar 15;12(3):694. doi: 10.3390/cancers12030694 (PMC7140077; doi:10.3390/cancers12030694)
Supplement: Supplementary file 1 [file cancers-12-00694-s001.pdf]

Supplementary Materials: Establishment and Molecular Phenotyping of Organoids from the Squamocolumnar Junction Region of the Uterine Cervix

Yoshiaki Maru, Akira Kawata, Ayumi Taguchi, Yoshiyuki Ishii, Satoshi Baba, Mayuyo Mori, Takeshi Nagamatsu, Katsutoshi Oda, Iwao Kukimoto, Yutaka Osuga, Tomoyuki Fujii and Yoshitaka Hippo

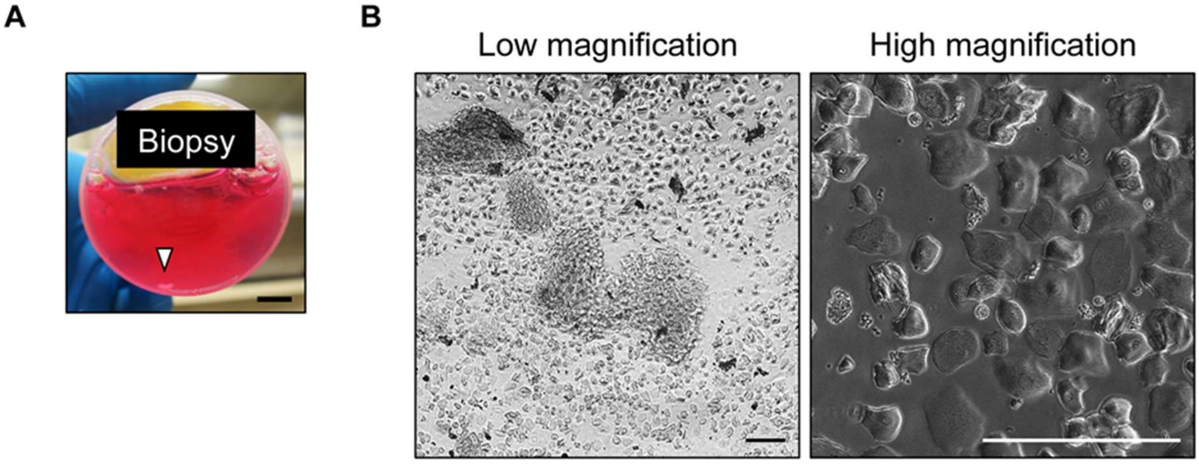

Figure S1. A failed case of organoid culture of biopsy samples. (**A**) Biopsy specimen of SCJ. (**B**) Phase contrast images of SCJ cells. Note that most cells appear flat and are likely superficial squamous cells. Scale bar indicates 200 μm.

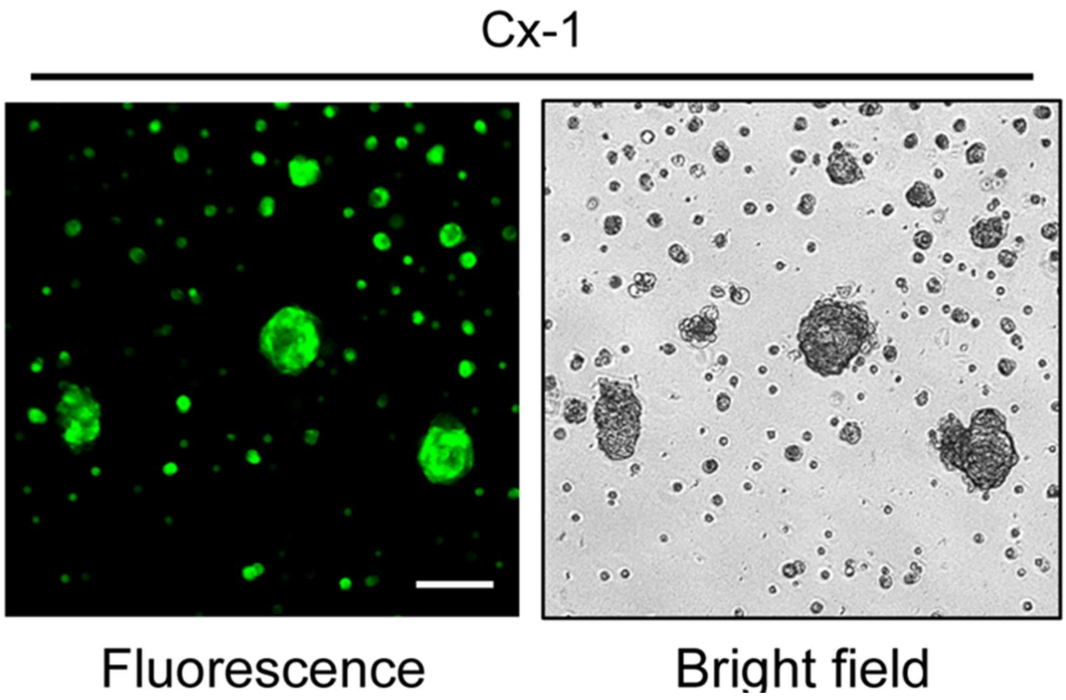

Figure S2. Highly efficient gene transduction of SCJ-derived organoids. Fluorescent and phase contrast images 2 days after introduction of a GFP-expressing vector into Cx-1organoids are shown. Note that the majority of the cells readily turned green without drug selection for GFP. Scale bar indicates 200 μm.

A

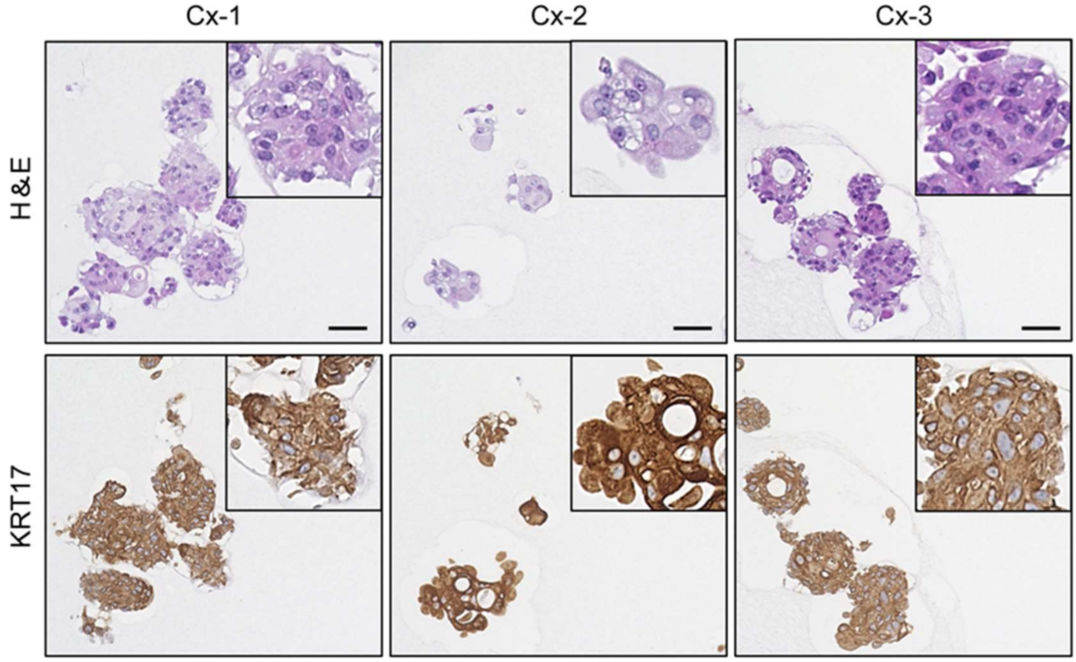

B

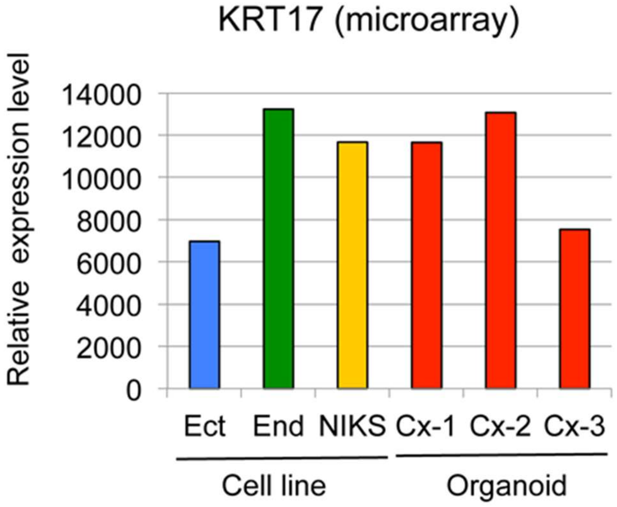

Figure S3. KRT17 expression in organoids. (**A**) Histological examination of thin sections. Organoids Cx-1 to Cx-3 were analyzed. Upper panel, H&E staining. Lower panel, immunostaining for putative reserve cell marker KRT17. Insets show magnified images. Scale bars indicate 50 μm. (**B**) Expression level of *KRT17* in microarray analysis.

Table S1. SCJ marker genes whose expression was not verified in organoids.

Low Expression	No Probe
CP	*C20orf114*
SLC34A2	*PLUNC*
PRKAR2B	*PLA2G10*
GLYATL2	*PPM2C*
HGF	*FLJ26056*
DPH3	*KCTD14*
UNC119B	*LOC388152*
HIST1H3A	*FLJ46210*
FGB	*CXorf52*
ANKRD36B	*PMS2CL*
C9	*NXF2*
OCLM	*MOP-1*
SPAG11B	
BICD1	
OR4P4	
SLC45A4	
LCE2D	

Among 77 genes reported as SCJ markers in a study by Herfs et al [1], genes with an average intensity less than 20 in both cell lines and organoids were excluded from Figure 3A. Genes with no matching probes in the microarray used in this study were also eliminated from Figure 3A.

Table S2. Primers for RT-qPCR.

Gene	Sequence (Forward/Reverse)	Product Size (bp)	Annealing Temperature (°C)	Extension Time (s)
GAPDH	5'-GAAAGGTGAAGGTCGGAGTC-3'<br>5'-GAAGATGGTGATGGGATTTC-3'	227	55	18
KRT7	5'-CAGGAACTCATGAGCGTGAA-3'<br>5'-AGAAGCTCAGGGCATTGCT-3'	205	63	9
AGR2	5'-GTCAGCATTCTTGCTCCTTGT-3'<br>5'-GGGTCGAGAGTCCTTTGTGTC-3'	97	59	4
MMP7	5'-GAGTGAGCTACAGTGGGAACA-3'<br>5'-CTATGACGCGGGAGTTTAACAT-3'	158	60	7
CD63	5'-GTAGCCCCCTGGATTATGGT-3'<br>5'-CTTGCTCTACGTCCTCCTGC-3'	108	58	5
GDA	5'-GCTGGAAGTAGCATAGACCTGC-3'<br>5'-TCTTCTGCAAAGTCGATGTTCTG-3'	95	58	4

References

1. Herfs, M.; Yamamoto, Y.; Laury, A.; Wang, X.; Nucci, M.R.; McLaughlin-Drubin, M.E.; Munger, K.; Feldman, S.; McKeon, F.D.; Xian, W.; et al. A discrete population of squamocolumnar junction cells implicated in the pathogenesis of cervical cancer. *Proc. Natl. Acad. Sci. United States Am.* **2012**, *109*, 10516–10521, doi:10.1073/pnas.1202684109.

© 2020 by the authors. Licensee MDPI, Basel, Switzerland. This article is an open access article distributed under the terms and conditions of the Creative Commons Attribution (CC BY) license (http://creativecommons.org/licenses/by/4.0/).
